# Foreign Psychological Literature

**Published:** 1860-07-01

**Authors:** 


### FOREIGN PSYCHOLOGICAL LITERATURE.

Our retrospect of Foreign Psychological Literature will embrace the following subjects:—

1. On a Form of Paralysis of the Lower Extremities, prevalent in part of the District of Allahabad.
2. On Puerperal Insanity.
3. On a Form of Cachexy peculiar to the Insane.

### 1. *Notice of a Form of Paralysis of the Lower Extremities, extensively prevailing in part of the District of Allahabad, produced by the use of Lathyrus sativus as an article of food.* By James Irving, M.D.

[The following paper is a highly-interesting and important contribution to our knowledge of the physical degenerations of the human species]:—

In October, 1856, Mr. Court, the Collector of Allahabad, when in Pergunnah Barra, on the right bank of the Jumna, was very forcibly struck by the number of lame persons whom he met in all directions. On inquiry he found, in village after village, that there were several cripples in each. He was also informed that the disease which gave rise to this lameness was of recent origin, and that it was attributed by some of the people to their living on bread made from *kessaree dâl*, and by others of them to the unwholesome qualities of the wind and water of the Pergunnah; the latter being vague causes of disease ever ready to be brought forward by the natives in order to account for any unusual or unintelligible sickness. Several cases of paralysis of the lower limbs were sent from Barra to the Government Charitable Dispensary at Allahabad, for medical treatment. Unfortunately, however, they got tired of the means employed for their cure, and left after being in hospital for a month or five weeks. But, through the kindness of Mr. Court, who accompanied me to Barra, I was enabled to make some few inquiries into the nature and history of the malady.

Close to the village of Kheerut Gohanee, on the Sohagee Road, all the lame people from surrounding villages were mustered for my inspection on the morning of the 6th February, 1857. About fifty men were present, all more or less lame in both legs; some so much disabled as to be hardly capable of motion, while others were only slightly affected. One after another was questioned, and the following particulars were thus gathered. Without exception they all stated that they had become paralytic during the rains; in most cases suddenly so; and several stated that it had been during the night. Men who had gone to bed quite well had awoke in the morning feeling their legs stiff and their loins weak, and from that day they had never regained the use of their limbs. At first, the lameness was trifling, and amounted only to unsteadiness of gait, and slight stiffness chiefly of the knees. After a time the muscles of the thighs commenced to ache and feel weak, and also the loins. In no case did those examined admit that they had then, or ever had, severe pain either in their limbs or loins. They all ascribed their disease to their feeding principally on *kessaree dâl*, but they seemed to imagine that, in order to produce the malady, there must be another circumstance superadded—viz., the deleterious quality of the water during the rains. So far as could be gathered, it was not from drinking the water that they fancied they took harm, but from getting wet by it. More than one dwelt on the fact of his having been exposed to rain, either while ploughing or tending sheep; and others spoke of having been working in jheels just before they became lame, at various periods embraced between the months of July and October. The people were particularly examined, and questioned as to whether they had had any symptoms of fever, or of any other disease at the time that they lost the use of their limbs, but they all said that they had not, and nothing was discovered to lead to the inference that this was not strictly true. In only one of many cases examined was enlargement of the spleen observed. Many of the men appeared to be strong-looking, and their legs even, in most cases, did not seem to be much wasted, if at all so. It was stated by those affected, as well as by several native officials who were interrogated on the subject, that the complaint did not lead to other diseases, nor tend to shorten life, unless indirectly by preventing the individual working, and thus procuring proper means of support. It was further stated, that the arms were never affected; but that there were some few cases of persons so greatly crippled that they could not walk. It was added, that males were more often afflicted than females; and that ryots were more liable to the disease than the zemindars, although the latter class was not exempt from it. . . . .

Now it appears, from a return with which Mr. Court kindly furnished me, that, in the month of January, 1857, there were, in Pergunnah Barra, 2028 persons known to be affected with paralysis, or in the proportion of one in every 31·30 of the population. These figures disclose a terrible amount of removable disease, showing a proportion of 3·19 per cent. of the population rendered useless by a single disease; for only a very few of those paralysed are able to do any work in order to support themselves.

To show further the extent to which palsy of the lower limbs prevails in Barra, the following facts may be stated. In the beginning of 1857 there were 295 villages, and I had a return showing that there were paralytics in 188 of these, thus leaving only 107 villages, scattered over an area of 158,493 acres, in which there were no cripples. But, of the 107 villages in which there were no cripples, there were 58 uninhabited, so that in reality there were only 49 inhabited villages in the whole pergunnah which were free from this species of paralysis.

Different villages, however, are affected in very different degrees and proportions. Thus in Kuchra, with a population of 371, there was only one paralytic, while in Soondurpore, with a population of 250, there were 39. In Puchiour, with a population of 375, there was one. In Buschora Uperhar there were 353 inhabitants, of whom 33 were paralysed. In the village of Abheepoor there were, in January, 1857, 268 inhabitants, of whom 22 were palsied. In Lohera, out of 557 villagers, 8 were cripples, or one in 69. In Pooreh Gunga Chuk there were 261 inhabitants, of whom 20 were palsied, or one in every 13. In Buradeh Zuptee, there were 148 inhabitants, 8 of whom were affected, or in the proportion of one in 18. In Room there were 6 paralysed villagers of 198, or one in 33. In Buckla there was a population of 491, and only 4 lame, or one in 122.

The disease, as might be expected, is not confined to Barra, but extends to Khairagurh, in Allahabad District, and to the adjoining District of Banda. Mr. Mayne, the Collector of the latter, supplied the following table and memorandum in reference to this malady :—

| Pergunnah. | Thannah. | No. of villages in which cripples are known to exist. | No. of cripples. | Population of Pergunnah. | Percentage. |
|---|---|---|---|---|---|
| Chiboo | Mow, | 23 | 64 | 80,170 | 0·249 |
| | Burgah, | 26 | 131 | | |
| | Rajapoor, | 3 | 5 | | |
| Total | ... | 52 | 200 | ... | ... |
| Enohan | Kurwee, | 10 | 14 | 82,313 | 0·139 |
| | Munikpoor, | 23 | 72 | | |
| | Bimree, | 12 | 34 | | |
| Total | ... | 45 | 120 | ... | ... |

"The greatest amount of cripples in pergunnah Chiboo, is to be found in Burgah, which is composed entirely of villages in the hills. In Mow, where the country is less hilly, the numbers decrease, and in Rajapoor, where there are no hills, we have none at all. The causes are given as gatheea, baiee, gurhun, beeadh, shukembad, gutteea, adhung. The same remarks apply to pergunnah Enohan. Thannah Munikpoor is entirely in the hill country, and the cripples are there more numerous. Thannah Bimree is less hilly, and has less cripples. Kurwee is in the flat country, and contains hardly any cripple."

When Mr. Mayne says that the causes are given as gutteea, &c., I presume he alludes to the native names by which this particular kind of palsy is known in the Banda District.

Before alluding to the cause of this form of paralysis, it may be as well to glance at the physical aspect of Barra, and the circumstances connected with it likely to produce sickness. In passing through this part of the country, it appears a vast swamp. One is struck not only by the great number of jheels, but also by the numerous tanks that are visible in all directions. These tanks, moreover, have generally one side, or part of a side, level with the surrounding ground, and are intended to drain the contiguous fields and render them arable. There are several low ranges of hills covered with large blocks of stone. The village of Barra stands high, and I was told that, looking down from it in the rains, nothing is visible but one vast sheet of water on all sides. This was the case so late as the month of December, in the year 1856. The soil of Barra is a stiff marl. It appears to take up water readily, and to retain it for a long time. In the hot weather it dries and splits into deep and wide fissures. I examined numbers of bricks made in different parts of the pergunnah, and found that none of them had the ring of good brick, when struck. They were easily broken, and a fracture generally showed numerous small calcined masses, chiefly of lime. There is a strong saline impregnation of the soil which shows itself by efflorescence on the surface. Lime made with the water soon crumbles away.

In March, 1856, some of the water from a well which was said to be poisonous to any animal that drank of it, was sent to the Chemical Examiner to Government for analysis, in consequence of a law-suit then pending in reference to the closing of the said well, on account of the deleterious qualities of its water. He reported that the water contained "no absolutely poisonous ingredient, but it holds in solution so large a quantity of saline matter that it would prove very deleterious to any animal habitually drinking it." He further stated that the saline ingredients consisted of Sulphates, Carbonates, and Chlorides of Lime, Magnesia, and Soda. Water is found very close to the surface. In several wells examined in February, it was only six feet, and in the rains it is said to rise within one foot of the surface of the ground.

The people of Barra appear to be very poor, and signs of their poverty are everywhere visible. The villages look dilapidated, and many of the houses are unoccupied. I saw no horses nor camels,—not even a common bullock cart. The bullocks that one does see, ploughing or carrying loads, are wretched, half-starved looking animals. The area of Barra is 158,493 acres, and the population 63,490, which gives an average of 256 to the square mile. But the general population of the North-West Provinces, according to the last Census, is at the rate of 420 to the square mile, and in the district of Allahabad generally, it is 493 to the square mile.

As has been stated, the paralytic symptoms which prevail so extensively in Barra are, by the natives, very generally attributed to their making large use of *kessaree dâl*,—the *Lathyrus sativus* of English botanists; and it is perhaps one of the most remarkable circumstances connected with the malady, that the people should be so fully persuaded that in eating this grain they eat poison, and that yet notwithstanding they have continued, and will continue to do so, from generation to generation.* *Kessaree dâl* is not unlike gram, and is common enough

* Mr. Court, Collector of Allahabad, in a letter to Government on this subject, writes:—"It will be an object with me to discourage as much as possible the cultivation of Kessaree Dâl, and the employment of that vetch as food. But I fear that this will have but little effect on the people. They all ascribe the disease to eating it, and yet they live upon it. The fact is they cannot help themselves. They must either eat it or die of starvation. In the highlands of the pergunnah, wheat will not grow; cotton is grown in the khareef; gram and linseed in the rubbee; but the staple produce of khareef harvest is the kodoo; of the rubbee, kessaree. There are other causes for this besides that of the soil being unsuited for wheat, &c. The pergunnah is particularly liable to disaster. Too much rain is as destructive to the better description of crops as too little, and as in that as in in most parts of India. It is frequently sown along with wheat or barley and cut green as fodder for cattle. In Barra the *kessaree dâl* is ground and made into bread. It is sometimes mixed with other grains, such as barley; but is more generally taken alone, the people, in fact, not being able to afford anything else. It is the cheapest grain procurable, and forms the chief support of the people from March till October. On the 7th February, 1857, in the bazaar at Barra, wheat sold at the rate of fourteen seers to the rupee, while *kessaree dâl* was at the rate of twenty-two per rupee. It grows without labour or trouble, and on damp swampy ground that will bear no other crops. The land is merely ploughed slightly once, and the seed thrown in; or the plant sows its own seed, which germinates freely next year without further attention or care. The moist nature of the soil of Barra should be noted in connexion with the production of this poisonous *Lathyrus;* for it is stated by Loudon—in speaking of *Lathyrus cicera* causing paralysis of the lower limbs, in those who live on bread partly made of it, in some continental states—that the plant grown on a strong moist soil is more injurious than that cultivated on one which is dry and light.*

That use of *kessaree dâl* as an article of food is apt to lead to paralysis of the lower limbs appears to be very generally known to the inhabitants of many parts of India. Dr. K. W. Kirk, in his *Topography of Upper Sindh*, says:—

"My attention was first attracted to it [Paralysis] as follows: a villager brought his wife, a woman of about thirty years of age, to my hospital, with paralysis of her lower extremities; she had been so afflicted for the last four years. I asked whether she had had a fall or a blow to cause the disease. 'Oh,' said the man, 'it is from *kessaree;* we are very poor, and she was obliged to eat it for five months on end!' I had never heard of such effects before from any grain, and asked whether it was good of its kind. Finding it was so, I sent the man into the bazaar to bring me a handful, which I afterwards showed to some respectable natives, and was told that disease from its use is very common all over the country. The villager above alluded to said, that if they had sowed a better kind of grain it would have been plundered by the Belooches from the hills, but they would not take this. I did not enter a village in Sindh where this *kessaree* was not to be found in the bazaar, and daily used by great numbers of poor people, nor where several were not rendered most helpless objects by the use of it. Their general health seemed good, however, their only complaint being that they had no power in their legs, but they moved about by lifting themselves on their arms. All natives know that this *dâl* is a poison, and eat it only because it is cheap, thinking that they can in time save themselves from its consequences."

Colonel Sleeman states, that in part of the Saugor Territories in 1829, and two succeeding years, the wheat crop failed from various reasons, and during these three years the *kessaree* remained uninjured, other pergunnahs bordering on the hills, hail-storms are very frequent. The kessaree grows in all seasons. It requires little or no culture; it varies in luxuriousness of growth only according to the season, and affords the only certain provision of life. As the people are at present circumstanced, they have, in real truth, no option.

* *Encyclopœdia of Plants*, p. 620. *Taylor on Poisons*, p. 536.

and thrived with great luxuriance. In consequence it formed the only food of the people during the three years of famine. "In 1831, they reaped a rich crop of it from the blighted wheat fields, and subsisted upon its grain during that and the following years, giving the stalks and leaves only to their cattle. In 1833 the sad effects of this food began to manifest themselves. The younger part of the population of this and the surrounding villages, from the age of thirty downwards, began to be deprived of the use of their limbs below the waist, by paralytic strokes; in all cases sudden, but some more severe than others. About half of this village, of both sexes, became affected during the year 1833-34, and many of them have lost the use of their lower limbs entirely, and are unable to move. Since the year 1834 no new case has occurred, but no person once attacked had been found to recover the use of the limbs affected." He further adds, that "many of those he saw were fine-looking young men, of good caste and respectable family. They stated that their attack had come on suddenly, often while the person had been asleep, and without any previous warning. Males were said to be more subject to the disease than females. They believed that both horses and bullocks fed on *kessaree* lost the use of their limbs."—(*Sleeman's Rambles and Recollections of an Indian Official*, vol. i. p. 134.) Dr. Thomas Thomson also, in his book of Travels in the Himalayas, mentions instances of paralysis caused by the use of *Lathyrus sativus* which he had observed in Thibet. I received the following information as to the prevalent ideas of the people of this part of the country in reference to *kessaree dâl* from a very intelligent educated native, the late Pursidh Narain Sing, a teh-seeldar in the Allahabad District.* The lameness, he writes, that results from the use of *kessaree dâl*, is supposed by the natives to be a mixture of palsy and rheumatism. Living on this particular grain is supposed to be the predisposing, and exposure to cold, rain, and damp weather, the exciting cause of the disease. He adds, "the bhoosa (or chaff) of this grain may be given to cows and bullocks without harm, but such is not the case with horses, who are affected (in consequence of eating it) with what is called by natives *koorkooree*. I do not know the English term for it. He describes the symptoms of this complaint in the horse, and both from the name he gives above, and from the description, there is little doubt that he alludes to colic, or gripes, an affection likely enough to result from this or any other indigestible food, and which would result independent of any specific action of this grain on the horse. Colonel Sleeman, as before noted, had been informed that *kessaree dâl* caused loss of power of the limbs in both horses and bullocks. Pursidh Narain Sing further informed me, that "the natives consider *kessaree dâl* to be void of all nourishment, and they declare it to have a peculiar effect on the lower part

* This man was Tehseeldar of Hundya, and was one of the *very very* few native officials at this place who, *really* and *in earnest*, stuck to us during the rebellion. He did so from the very first to the very last. For having gone to Cawnpore along with General Neil, he was sent to Bithoor with a party of Mehter police for the purpose of re-establishing order, and was there attacked by a large band of rebels, and cruelly put to death.

of the spine. It is also said that *kessaree* grain makes the system susceptible of catching other diseases, such as scrofula, particularly in Patna District."

In Europe also, paralysis of the lower limbs has been observed to follow the use of *Lathyrus sativus* as an article of food. Thus Don, in the Gardener's Dictionary, says, that the flour of this plant, mixed with wheat flour in half the quantity, makes very good bread, but alone produces surprising rigidity of the limbs in those who use it for a continuance. In the same quarter of the globe similar effects have also been observed to follow the eating of other kinds of grain produced by the same great natural order of plants, the Fabiaceæ—to which the *Lathyrus sativus* belongs; as well as other species of the same genus. Thus Dr. Taylor alludes to *Lathyrus cicera* and *Ervum Ervilia* (bitter vetch) as occasionally rendering bread poisonous. In some part of the Continent, a bread is made from the flour of the *Lathyrus*, which is so injurious in its effects, that the use of it has frequently caused its prohibition by law. Loudon states, that when mixed in equal parts with wheaten flour it makes a good-looking bread, which, however, occasionally gives rise to weakness of the knees and spasmodic contractions of the muscles. Cattle and birds, when fed on the seeds, are said to become paralysed. A more recent example of the poisonous effects of *Lathyrus cicera* flour is furnished by M. Vilmorin; he remarked that "the use of this bread for a few weeks produced complete paralysis of the lower extremities in a young and healthy man. Six or seven individuals of the same family who had eaten it suffered more or less from similar symptoms, and one had died. A physician who practised in the district remarked that paralytic affections were very common among the poor, who subsisted on this bread, while they rarely occurred among the better classes. When the *Lathyrus* flour formed one-twelfth part, no inconvenience was observed to attend its use; in a proportion greater than this it becomes injurious; and when it amounted to one-third part, the effects might be serious." (Ann. d. Hyg. Avril 1847, p. 469—Taylor on Poisons, p. 536.) Dr. Lindley also states, that the seeds of *Ervum Ervilia*, mixed with flour and made into bread, produce weakness of the extremities, especially of the lower limbs, and render horses almost paralytic. (*Vegetable Kingdom*, 2nd Edit. p. 548.)

As to the treatment of cases of paralysis caused by the use of *Lathyrus sativus*, I have little to say from practical experience. About a dozen cases have come under my observation at the Dispensary, but most of them disliked the restraint and the means of cure employed, and left after they had been patients for a month or five weeks. In some strychnine was tried; in others blisters to the loins frequently repeated; in others tonics. To all I gave generous diet. Two seemed to be somewhat benefited, and could walk better; and in one case the improvement was such, that a man who formerly could only walk with the aid of two sticks could after a time proceed without any assistance. He was under treatment at the time of the rebellion in June, 1857, when the Dispensary was burnt down by the "*poor natives*," for whose use it had been built and maintained by Government. What seemed to me of most use were tonics and generous diet, together with the application of occasional blisters.

The natives of Barra do not appear to have any kind of rational treatment. They rub the lower extremities with various liniments, of which one is composed of oil, garlic juice, and opium. They fancy that eating pigeon's flesh is of use. It was stated to Mr. Court that this affection was of recent origin in Barra; but on asking a native official who had known the purgunnah for twenty years past, I was informed that the disease had, to his knowledge, always existed; although he thought that of late it had become more common; and villages in which formerly there were no cripples now contained several. —*Indian Annals of Medical Science*, July, 1859.

---

2. *Observations upon Puerperal Insanity.* By RICHARD GUNDRY, M.D., Assistant Physician to the Southern Ohio Lunatic Asylum.

[In a very able and interesting paper, Dr. Gundry has elaborately analysed fifty-six cases of puerperal insanity, and compared the results of his own experience and research with those recorded by other observers. He divides the puerperal state into—

1. The period of gestation; including both conception and delivery.
2. The period extending about two months from delivery, during which the involution of the uterus is completed, and the function of lactation is thoroughly established.
3. The period of lactation, including weaning and the changes induced by the decline and cessation of the lacteal secretion.

The following important data are extracted from this paper:—]

*Proportion of Cases of Puerperal Insanity in Asylums.*—In an analysis of the causes of insanity in 11,762 insane women, reported from fourteen hospitals for the insane in the United States, 1050 are noted as occurring during the puerperal period; or nearly 1 in 11 insane females. The reports of foreign hospitals for the insane would doubtless tell the same story. During five years one-eighth of the females admitted into Bethlem (London) were subjects of puerperal insanity. At Salpêtrière a twelfth, and during some years a tenth, were of the same nature. The experience of these two metropolitan hospitals is thought by Dr. Tuke to be above other institutions. He estimates that in most English asylums one-fourteenth to one-twentieth of the females admitted is the proper proportion. The results of a careful examination of the cases given above do not enable me to concur with this opinion, but exactly agree with the deductions from the experience of Bethlem and Salpêtrière, and it may be questioned whether they represent fully the proportion chargeable to this cause. Esquirol met in private practice with a still greater relative number of cases, and this has been the experience of several eminent practitioners who have had abundant opportunities for observation. From various sources we derive the following statistics on this subject.

| | Number of Insane Females. | Puerperal Cases. |
|---|---|---|
| 14 American Asylums . . . . . . . . . . . | 11,762 | 1050 |
| Reported by Dr. McDonald . . . . . . . . . | 691 | 49 |
| " " M. Parchappe . . . . . . . . . | 596 | 33 |
| " " M. Seller . . . . . . . . . . . . | 97 | 11 |
| " " Hanwell Asylum . . . . . . . . | 703 | 79 |
| " " M. Mittivie . . . . . . . . . . | 242 | 9 |
| " " M. Esquirol . . . . . . . . . . | 1119 | 92 |
| " " Bethlem Hospital . . . . . . . . | 899 | 111 |
| Totals. . . . . . . . . . . | 16,109 | 1434 |

According to which table, of every 100 insane women, nearly 9 became so in consequence of the puerperal condition in some of its stages. On the other hand, the records of lying-in hospitals show that a very small proportion of the whole number of women *confined* become insane. In the Westminster Lying-in Hospital, according to Dr. Reed, only 9 out of 3500 delivered there were attacked. In Queen Charlotte's Lying-in Hospital, in 2000 cases there were eleven who became insane. Other institutions of a similar nature furnish like results. It must be recollected, however, that the time spent in a lying-in hospital after delivery is usually very short, and does not include the period of lactation most productive of mental disease.

*Analysis of Fifty-six Cases.—Age.*—The age at which insanity first appeared in these cases, was as follows:—

| | Cases. | Ratio. |
|---|---|---|
| Under 20 years of age . . . . . . . . . . . . . | 3 | 5·36 |
| " 25 " " . . . . . . . . . . . . . | 18 | 32·14 |
| " 30 " " . . . . . . . . . . . . . | 11 | 19·64 |
| " 35 " " . . . . . . . . . . . . . | 13 | 23·21 |
| " 40 " " . . . . . . . . . . . . . | 8 | 14·29 |
| " 45 " " . . . . . . . . . . . . . | 3 | 5·36 |
| Totals . . . . - . . . . . . . | 56 | 100. |

It will be seen that from 20 to 25, and from 30 to 35, the proportion is much larger than at any other period; but this might have been expected, so far as the first period is concerned; for the proportion of females living at that age in the United States greatly exceeds that of any other period, excepting less than 20 years of age. So far as any conclusion can be drawn from such limited data, it points to the period of life between 30 and 35 as the time most prolific of puerperal insanity. Many of the cases in our survey did not come under observation during the first attack. We must therefore inquire the age at which the attack (herein alluded to) was developed:—

| | | | | |
|---|---|---|---|---|
| Between 20 and 25 in | 9 cases. | | Between 40 and 45 in | 3 cases. |
| ,, 25 ,, 30 ,, | 15 ,, | | ,, 45 ,, 50 ,, | 2 ,, |
| ,, 30 ,, 35 ,, | 16 ,, | | | — |
| ,, 35 ,, 40 ,, | 11 ,, | | Total . . | 56 |

And in this connection we must also take into account the number of attacks suffered. Thus, it was—

| | | | | |
|---|---|---|---|---|
| The 1st attack in | 37 cases. | | The 4th attack in | 4 cases. |
| ,, 2nd ,, ,, | 10 ,, | | No. unknown ,, | 1 ,, |
| ,, 3rd ,, ,, | 4 ,, | | | — |
| | | | Total. . . | 56 |

We shall more full understand the influence of age by ascertaining the periods at which each of these attacks occurred :—

| Periods. | Cases of one Attack. | Of two attacks. | | Of three attacks. | | | Of four attacks. | | | | Total No. Attacks. |
|---|---|---|---|---|---|---|---|---|---|---|---|
| | | 1st | 2nd | 1st | 2nd | 3rd | 1st | 2nd | 3rd | 4th | |
| Less than 20 years. . | 0 | 2 | | 1 | | | | | | | 3 |
| ,, ,, 25 ,, . . | 6 | 6 | 2 | 2 | 2 | 1 | 3 | 3 | | | 25 |
| ,, ,, 30 ,, . . | 9 | | 2 | 1 | 1 | 1 | 1 | | 3 | 3 | 21 |
| ,, ,, 35 ,, . . . | 11 | 2 | 2 | | 1 | 1 | | 1 | 1 | 1 | 20 |
| ,, ,, 40 ,, . . | 8 | | 3 | | | | | | | | 11 |
| ,, ,, 45 ,, . . | 3 | | | | | | | | | | 3 |
| ,, ,, 50 ,, . . | | | 1 | | | 1 | | | | | 2 |
| Totals. . . . . | 37 | 10 | 10 | 4 | 4 | 4 | 4 | 4 | 4 | 4 | 85 |

The history of the 55 persons, therefore, embraces 85 different attacks of insanity, of which more than one-half occurred between 25 and 30 years of age; while taking those only who had one attack, the period from 30 to 40 furnishes more than one-half. Whether the inference to be drawn from this, that those attacked more early are more liable to a recurrence of the disease, is warranted by the other circumstances of the cases, will afterwards be adverted to. One case of the 56 not included in the above analysis had suffered from several attacks (the exact number not being known to me), of which the first took place before 25 years, and the last at 32 years of age; from all of which she perfectly recovered.

*Occupation.*—The influence of occupation receives but feeble illustration from this series of cases. All classes seem equally liable. Neither riches, with the luxury that attends, nor poverty, with its supposed exemption from enervation, can claim any exemption.

*Civil Condition.*—As to their civil condition very little can be said. 54 patients were married, and 2 single women. Esquirol remarked that the number of single persons becoming mothers, who are afflicted with puerperal insanity, bears a large proportion to the married. Of 92 cases reported by him, 63 were married and 29 single. We might expect, *à priori*, that if moral causes exerted so preponderating an influence in the production of insanity as many writers assert, a larger number of those unfortunate women who have borne illegitimate offspring would be found subjects of this disease than the statistics of insanity in any country exhibit.

*Hereditary Transmission.* — How far does hereditary tendency display itself in cases of this description? This is a difficult question to answer correctly, for no point is more assiduously concealed by the friends of parties than the existence of any hereditary taint. Where collateral relatives have been insane, I have included them in my estimate, as leading to the surmise of a taint in the common ancestry, in the absence of precise information; though such evidence is by no means conclusive:—

| | | |
|---|---|---|
| Father had been insane | in | 3 cases. |
| Mother ,, ,, ,, | ,, | 6 ,, |
| Father, brother, and 6 sisters | ,, | 1 ,, |
| Mother, and mother's sister | ,, | 1 ,, |
| Great-grandfather (father's), and sister | ,, | 2 ,, |
| Brothers or sisters | ,, | 4 ,, |
| Father's brother | ,, | 1 ,, |
| Uncle and aunt | ,, | 1 ,, |
| Hereditary (relationship not specified) | ,, | 3 ,, |
| Not ascertained | ,, | 34 ,, |
| Total | | 56 |

Twenty-two out of fifty-six, or two in every five, are suspected or known to have been predisposed to mental disorders by the existence of hereditary taint. This corresponds with the proportion observed by Esquirol, while Dr. Burrows found, in 80 women who became insane after delivery, more than half hereditarily predisposed. Dr. Gooch remarks: "A very large proportion occurred in patients in whose families disordered minds had already appeared;" and in 217 cases collected by Helfft, Weill, and Marcè, 89, or 41 per cent., belonged to this class.

*Unusual Circumstances.* — Any unusual circumstances affecting the patient about the time of the attack must be taken into consideration, as exercising more or less influence in its causation. Of such several have been ascertained in this series of cases. These can be arranged as follows:—

| | | | |
|---|---|---|---|
| Lost a child prior to last delivery | 3 | Chorea | 1 |
| Miserably situated at confinement | 2 | Abscess of breasts | 1 |
| Drunken or worthless husband | 5 | Illegitimacy of child | 3 |
| Inflammation of uterus | 3 | Repelled papular eruption | 1 |
| Leucorrhœa | 1 | ,, ulcer | 1 |
| Still-born child | 1 | Mental emotion | 1 |
| Family difficulties | 2 | No unusual circumstances | 26 |
| Child ruptured (grief) | 1 | Not ascertained | 4 |
| Total | | | 56 |

*Primiparæ and Multiparæ.*—Are primiparæ more liable to become affected than multiparæ? Among 53 persons (3 being unknown), it was observed there were attacked in connection with the—

| | | | |
|---|---|---|---|
| 1st labour | 10 | 3rd and 4th labours | 1 |
| 1st, 2nd, and 3rd labours | 3 | 4th ,, | 6 |
| 1st and 3rd ,, | 1 | 5th ,, | 3 |
| 1st, 2nd, 3rd, and 4th ,, | 3 | 5th, 6th, 7th, and 8th labours | 1 |
| 2nd ,, | 8 | 7th ,, | 2 |
| 2nd and 3rd ,, | 1 | After every labour (No. unknown) | 1 |
| 3rd ,, | 9 | After having borne several children. | 4 |

Only 18, or 1 in 2·94 of the number were primiparæ; while of these 7 had a repetition of the attack in connection with every child born to them. When insanity has once established itself as one of the incidents of the puerperal condition, it seems to have a great tendency to appear at every successive period of that kind. This periodicity was noticed in 10 women, and was established in the 1st, the 2nd, the 3rd, and even the 5th puerperal state. In only one case did it skip over one child after it had once appeared, and then re-appeared with the next. M. Marcè found in 57 patients only 14 primiparæ; and amongst the 43 remaining cases, 13 had been confined 5, 6, and even 9 times.

*Circumstances attending the Labour.*—How far the nature and history of the labour influenced the production of insanity which may have ensued, is open to much discussion. Difficult and tedious labours seem as innoxious as regular and easy labours. Nor are those who have flooded profusely more certainly liable to an attack than any others. Drs. Merriman, Gooch, Esquirol, Frias, Selade, Billod, and Reid, mention one instance each of insanity in connexion with labour, complicated with puerperal convulsions, and apparently dependent on that cause. Yet the proportion of patients with eclampsia becoming deranged is exceedingly small; too trifling to furnish evidence of any relation existing between them, as cause and effect. Dr. Webster has charged upon the use of chloroform in labour some few cases of puerperal insanity, and Dr. Skae, of the Edinburgh Asylum, reports one case attributed to this, the only one out of 44 cases of puerperal insanity admitted into that institution since the discovery of chloroform. On the other hand, Dr. Simpson relates two cases where the use of chloroform in labour prevented the expected usual attack of mania after it.

*Period of Invasion:*—

| Period of Attack. | 1st Epoch. | 2nd Epoch. | 3rd Epoch. |
|---|---|---|---|
| During pregnancy | 7 | | |
| Less than 15 days after delivery | | 20 | |
| ,, ,, 1 month ,, ,, | | 5 | |
| ,, ,, 2 months ,, ,, | | 4 | |
| ,, ,, 3 ,, ,, ,, | | | 2 |
| ,, ,, 4 ,, ,, ,, | | | 6 |
| ,, ,, 6 ,, ,, ,, | | | 4 |
| ,, ,, 12 ,, ,, ,, | | | 2 |
| Immediately after weaning (about 12 months after delivery) | | | 2 |
| Unknown, but during lactation | | | 4 |
| Totals | 7 | 29 | 20 |

To complete the history of this stage of the disease, it may be necessary to ascertain at what period the previous attacks already alluded to occurred. Four attacks were non-puerperal; four attacks were during pregnancy; fourteen attacks were during the second epoch; two attacks were during lactation; and the circumstances of *six** attacks are unknown. So that of 76 known puerperal attacks, 11 began during pregnancy; 43 during the two months following delivery; 22 during lactation, or immediately after weaning.

Compare with these the results of others. Esquirol thus classifies 92 cases:—

| | |
|---|---|
| From first to fourth day after delivery . . . . . . . | in 16 cases. |
| ,, fifth to fifteenth ,, ,, . . . . . . . . | ,, 21 ,, |
| ,, sixteenth to sixtieth ,, ,, . . . . . . . . | ,, 17 ,, |
| ,, sixtieth to twelfth month of suckling . . . . | ,, 19 ,, |
| After forced or voluntary weaning . . . . . . . . . | ,, 19 ,, |

A summary of the cases reported by Dr. McDonald, Dr. Burrows, M. Marcè, and at Hanwell, in 1848, and those collected by M. Marcè, and by Dr. Wyman, of Boston, in Report of 1834, may also be useful for comparison:—

| Period of Attack. | Dr. McD. | Dr. B. | M. M. | At H. | M. M. | Dr. W. | Total. |
|---|---|---|---|---|---|---|---|
| During Pregnancy . . . . . . . . | 4 | | 18 | 4 | 27 | 5 | 58 |
| After Delivery . . . . . . . . . | 44 | 51 | 41 | 26 | 180 | 28 | 370 |
| During Lactation . . . . . . . . | 18 | 6 | 20 | 13 | 103 | 47 | 207 |
| Total . . . . . | 66 | 57 | 79 | 43 | 310 | 80 | 635 |

The second period is, therefore, the most important in the causation of disease. Some have regarded that as embracing only six weeks after delivery instead of two months, the limit I have adopted; so that were the figures corrected to that period, the numbers of the second epoch would be still slightly increased.

*Access of Disease.*—The access of the disease was marked by symptoms in this series, which were, in many instances, doubtless but imperfectly recorded. Often the alterations of manner, of feeling, the emotional and instinctive changes which ushered in the attack, were either unobserved, or their value unappreciated. Something startling, out of the usual routine of life, must occur to be remembered as the starting point in the history of mental disease in such cases. I have estimated the first symptoms as closely as possible, in the following table:—

* These *six* attacks, marked as unknown, occurred either during the second or third epoch,—in which is unknown.

| Nature of the First Symptoms. | No. Cases. | Nature of the First Symptoms. | No. Cases. |
|---|---|---|---|
| Unfounded jealousy and suspicion | 4 | Quarrelsome propensity . . . | 1 |
| Fear of injury from persons . . | 3 | Attempt to kill child . . . . | 3 |
| Attempts to wander away . . . | 5 | Suicidal attempt . . . . . . . | 4 |
| Indifference to child . . . . . | 3 | Suicidal and homicidal attempt on children . . . . . . . | 1 |
| Excited talk . . . . . . . . | 9 | First symptoms not ascertained . | 8 |
| Delusions . . . . . . . . . | 7 | | |
| Hallucinations . . . . . . . | 4 | Total . . . . | 56 |
| Fear of impending evil . . . . | 3 | | |
| Ecstatic feeling . . . . . . . | 1 | | |

*Form of Disease.*—The form of insanity assumed by the patient is to some extent influenced by the epoch in which the attack began. This may be seen, so far as the 56 cases are concerned, in the following table:—

| Form of Mental Disease. | 1st Epoch. | 2nd Epoch. | 3rd Epoch. | Totals. |
|---|---|---|---|---|
| Mania . . . . . . . . . . . . . . . . | 5 | 22 | 9 | 36 |
| ,, periodical . . . . . . . . . . . | | 1 | | 1 |
| Melancholia . . . . . . . . . . . . . | 2 | 5 | 7 | 14 |
| Dementia (primary) . . . . . . . . . . . | | 1 | | 1 |
| Monomania of fear . . . . . . . . . . . | | | 2 | 2 |
| ,, ,, unseen agency . . . . . . . . | | | 1 | 1 |
| ,, ,, suspicion . . . . . . . . . | | | 1 | 1 |
| Totals . . . . . . . . | 7 | 29 | 20 | 56 |

Mania is the most frequent form, and especially predominates in the cases occurring soon after recovery. In those during lactation, melancholia, and those partial forms (more allied to melancholia than to mania), more nearly balance the number in which mania occurred. The experience of M. Marcè accords in assigning the highest number of cases to mania. All other authorities coincide. There is, however, a class of cases which rarely has any examples in any hospital for the insane, wherein mental disturbance (of the type of delirium rather than mania) supervenes a few days after delivery, and very rapidly terminates, either by recovery (as in the large majority) or in death. The proportion of such is difficult to be arrived at.

*Suicidal and homicidal Propensities.*—A distressing symptom, frequently met with in all forms of puerperal insanity of every epoch, is the perversion of the instinct of self-preservation. Attempts at suicide are often very suddenly put into force, and persevered in. It may be the first symptom of mental derangement that arouses the alarm of friends, as in the five cases already noted, or it may be developed at any time during the course of the attack. A lady during lactation, not previously deranged, was discovered by her husband hanging in the room at night; so quiet had been her movements, that he had not been awakened. She was resuscitated, and passed into deep melancholia, from which she gradually recovered, to all appearances, so that the vigilant oversight hitherto maintained, were relaxed. No sooner did this take place than again she hanged herself. Resuscitation, followed by a similar state of melancholia, was again succeeded by apparent restoration of cheerfulness and reason, when the suicidal impulse again suddenly re-appeared. The case is not embraced in this series, and the final result I am not acquainted with. In the 56 cases twelve attempted suicide, and three threatened or contemplated such a course. The manner of attempts may be noted:—

| Manner of Suicidal Attempt. | Mania. | | | Melancholia. | | | Monomania. | | | Total No. Cases. |
|---|---|---|---|---|---|---|---|---|---|---|
| | 1st Epoch. | 2nd Epoch. | 3rd Epoch. | 1st Epoch | 2nd Epoch. | 3rd Epoch. | 1st Epoch. | 2nd Epoch. | 3rd Epoch. | |
| By cutting throat . . . | | 1 | | | 1 | | | | | 2 |
| ,, drowning . . . . . . | | 1 | 1 | | | | | | 2 | 4 |
| ,, hanging . . . . . . | | | | | | 1 | | | | 1 |
| ,, several modes. . . . | | 1 | 1 | | 1 | 2 | | | | 5 |
| Totals . . . . | | 3 | 2 | | 2 | 3 | | | 2 | 12 |

In Bethlem Hospital, out of 111 cases of puerperal insanity 32 were affected by the suicidal impulse.

Allied with this perverted instinct, and appearing sometimes in the same individuals, is the propensity or impulse to kill. The victims selected are usually those naturally claiming the love and sympathy of the patient.

One patient tried to kill by scalding various persons ; three patients tried to kill husbands ; four patients tried to kill their infants ; one tried to kill children and husband; so that nine, or one in six developed homicidal propensities. Of these, three were combined with the suicidal impulse.

*Hallucinations, Illusions, &c.*—

| Nature of Symptoms. | Mania. | | | Melancholia. | | | Monomania. | | | Total No. Cases. |
|---|---|---|---|---|---|---|---|---|---|---|
| | 1st Epoch. | 2nd Epoch. | 3rd Epoch. | 1st Epoch. | 2nd Epoch. | 3rd Epoch. | 1st Epoch. | 2nd Epoch. | 3rd Epoch. | |
| Hallucinations of hearing . . . . | | 2 | 2 | | | 1 | | | | 5 |
| ,, ,, sight . . . . . | | 2 | | | | 1 | | | 1 | 4 |
| Illusions of hearing . . . . . . . | | | 1 | | | | | | | 1 |
| ,, ,, sight . . . . . . . . | | 1 | | | | | | | | 1 |
| Hallucinations of sight and hearing . | | | 1 | | | | | | | 1 |
| ,, ,, ,, hearing & smell | | | | | | | | | 1 | 1 |
| ,, ,, hearing, and illusions of sight . . . . . . . . . | | 2 | | | | | | | | 2 |
| Delusions . . . . . . . . . . . | 2 | 4 | 4 | | 2 | 2 | | | 1 | 15 |
| Totals . . . . . . | 2 | 11 | 8 | | 2 | 4 | | | 3 | 30 |

More than one-half, therefore, of the 56 cases were more or less under the control of some form of illusion. They heard voices directing them to do this, or to refrain from that. They saw robbers, or deceased relatives, or acquaintances, or faces constantly peering at them from the ceiling or the window. Several entertained that most harassing delusion that they were eternally lost, having committed the unpardonable sin: one that her husband had bewitched her, and another that she could cure all the sick by laying her hands upon them. In one case, ecstacy "rapt her soul in Elysium," but soon the scene changed to the blackness of despair, and a fear of demons who were tormenting her took possession of her.

*Prognosis.*—The prognosis of puerperal insanity is stated by writers in exceedingly favourable terms. The proportion of recoveries is quite large, according to the following authorities:—Dr. McDonald records that 80 per cent. of his cases recovered. Dr. Webster thinks that "three in every five cases of puerperal insanity may be confidently expected to recover within the year." Dr. Haslam cured 50 out of 85 cases at Bethlem. Dr. Burrows records 57 cases, of whom 35 recovered. Drs. Gooch and Prichard have considered these results as indicating a less favourable prognosis than the circumstances would justify, inasmuch as the cases are taken from hospital practice, and usually do not come under care under the most recent, and consequently curable stage of the disease. Dr. Gooch observes: "Of the patients about whom I have been consulted, I know only two who are now, after many years, disordered in mind, and of them one had already been so before her marriage."

So far as regards the present analysis, the following results are obtained:—

| | | | | |
|---|---|---|---|---|
| Recovered . . . | 31 persons | or | 55·35 | per cent. |
| Improved . . . . | 4 ,, | ,, | 7·14 | ,, |
| Died . . . . . | 6 ,, | ,, | 10·71 | ,, ,, |
| Were not improved. | 15 ,, | ,, | 26·78 | ,, ,, |
| Total . . | 56 | | | |

For the rest, of the 37 persons labouring under mania, 19 recovered, 3 improved, and 4 died, 11, after periods varying from eight months to ten years, remaining unimproved.

Of the melancholic, and other depressed forms of mental disease, including nineteen persons, twelve recovered, one improved, two died, and four had received no benefit, in periods of from four to ten years.

Of seven cases occurring during pregnancy, three recovered, one improved, and three were not improved.

Of 29 occurring within two months after parturition, fifteen recovered, three improved, three died, and eight received no benefit from treatment.

Of 20 cases occurring during lactation, thirteen recovered, three died, and four were not improved.

We have already seen that these 56 persons had previously suffered, in some instances, from one or more attacks of puerperal insanity. Taking the number of these attacks as the basis of our calculations, we have the following interesting results:—

| Period of Attack. | Recovered. | Improved. | Died. | Not Improved. | Totals. |
|---|---|---|---|---|---|
| Occurring during 1st epoch . . . . | 7 | 1 | | 3 | 11 |
| ,, ,, 2nd ,, . . . . | 29 | 3 | 3 | 8 | 43 |
| ,, ,, 3rd ,, . . . . | 15 | | 3 | 4 | 22 |
| ,, in 2nd or 3rd (which unknown) . | 6 | | | | 6 |
| Totals . . . . . . | 57 | 4 | 6 | 15 | 82 |

This would give a ratio of 69·51 per cent. of recoveries from attacks.

An interesting inquiry may arise, whether the chances of recovery are lessened by the previous occurrence of attacks in the individual? Some light will be gained by investigating the results of the attacks, embraced in the present analysis: —

| Number of Attacks. | Recovered. | Improved. | Died. | Not Improved. | Totals. |
|---|---|---|---|---|---|
| First attack . . . . . . . . . | 38 | 4 | 5 | 8 | 55 |
| Second ,, . . . . . . . . . | 14 | | | 4 | 18 |
| Third ,, . . . . . . . . . | 6 | | | 2 | 8 |
| Fourth ,, . . . . . . . . . | 2 | | 1 | 1 | 4 |
| Totals . . . . . . | 60 | 4 | 6 | 15 | 85 |

This is exclusive of one patient who had several attacks (the number not ascertained). We may safely assume two attacks, though three or four would probably be more correct. But *four* of the first attacks were non-puerperal in their character; deducting these, and adding the two as above, we shall have the following proportion of recoveries in 83 known puerperal attacks :—

| | | |
|---|---|---|
| Recovered from 1st attack . . . . . . | 35 out of 52 ; | or 67·3 per cent. |
| ,, ,, other than 1st attack . | 23 ,, ,, 31 ; | ,, 74·2 ,, ,, |
| Total . . . . . . . . | 58 out of 83 ; | or 69·8 per cent. |

Thus it would appear that the first attack is more disastrous to life and reason than when several attacks have been safely borne. If we take those who did not recover, or those who died, and make the same comparison, we shall arrive at the same result.

The influence of time upon the prognosis is shown in the general table of results. We may notice more particularly a few of the mcre important points in this connection :—

Within six months 13 recovered, and 1 died; in one year, 10 recovered, 3 died, and 2 did not improve; in 18 months, 4 recovered, and 1 did not improve; in two years 4 recovered, 1 died, and 1 did not improve; over 2 years, 4 improved, 1 died, and 11 did not improve.

*Treatment.*—No special means were resorted to. Such indications as from time to time appeared were met with appropriate treatment. In a majority of the cases, anodynes, in some form or other, entered into the medicinal treatment. Morphine and camphorated tincture of opium, were perhaps the most frequently called into requisition. Both in mania and melancholia they seem to relieve the tired brain from the fatigue of its own teasing vagaries, and, besides the inducing of sleep at night, exert a beneficial effect. Tonics are also generally required, and none are better than the various ferruginous compounds. The citrate, the tartrate, the muriated tincture may be used, as the taste of the physician or the special case would suggest. The carbonate combined with conium, "Brigham's mixture of iron and conium," with the occasional addition of morphine, answers admirably as a general tonic.

In the sleepless ravings of some cases, chloroform may be cautiously applied. In a few cases it was useful for the immediate purpose of inducing sleep, but further than this I have not observed any marked effect on the course of the disease. A more reliable course for a more permanent benefit to this class of patients, is the free administration of diffusible stimulants. Where opiates fail to induce sleep, and chloroform can only induce temporary quiet, these often act like a charm, in soothing irritation and producing good, refreshing sleep. Wine, spirits, ammonia, sulphuric ether, according to the special exigencies of the case, are thus beneficial.

Cathartics have always played a very conspicuous part in the treatment of insanity, puerperal and otherwise, ever since 'Naviga ad Anticyram' conveyed a reproach, as well as suggested the therapeutic means to put it away. But it may be doubted whether the practice has not often been carried too far. Their occasional use in this disease is undoubtedly proper, not because the patient is insane, but to procure relief when she is constipated. In the same way emetics may be occasionally useful. In both cases it is rather to remove causes of disturbance, than from any other reason.

In two or three cases I have seen great benefit from the continued use of quinine in moderate doses, with an occasional anodyne. They were of a low, nervous, melancholy character, with loss of appetite, inability to apply themselves to any object requiring attention, and a depressing, somewhat hysterical state of feelings.

In a word, the treatment of puerperal insanity is, to brace up the enfeebled body and shattered nerves, to procure as absolute quiet and repose for the organ of the mind as we gain for a broken bone by the use of splints. To devise the special means by which this end may be attained, constitutes the difficulty of treatment in the one case as in the other. And there is, in both cases, a point in their history, when, passive treatment having done its work, it needs to be replaced by action of the limb, or of the brain, as the case may be.

To recognise the exact time when this point is reached, and to make the change of means judiciously, should ever be objects of the greatest care. When mental exercise can be safely substituted for mental quiet (now passing into lethargy), excitement and of the emotions replaces indifference, then they are not only proper, but almost imperative. But, to be too hasty in this matter, is only to renew the former trouble. On the other hand, too long delay allows the patient to sink into partial fatuity. (*American Journal of Insanity. Jan.* 1860.)

---

3.—*On a form of Cachexy peculiar to the Insane.* By Dr. E. BILLOD, Chief Physician and Director of the Lunatic Asylum of Sainte-Gemmes-sur-Loire.

[IN a communication addressed to the Academy of Medicine in 1855, Dr. Billod made known the existence, in several French asylums, of a pellagrous affection which he held to be peculiar to the insane. The results of his subsequent researches upon the subject are summed up in the following paper.]

In order to complete our study of this subject, and to sum up the various opinions, it only remains for us to demonstrate the existence of a cachexy, which specially belongs to the state of insanity, of which the affection which we have hitherto described by the name of a *variety of pellagra proper to the insane, or pellagra consequent upon mental alienation* only, constitutes one of the forms—say the pellagrous form —and to consider it in general and under all its forms; such is the object of the present paper.

Although this general disposition of the health of the insane at certain periods of mental alienation has never yet been the subject of a special description, we do not believe that its existence will be disputed by any physician, however little he may be versed in the study of mental disorders, and we shall have no difficulty in gaining admission by the side of the various known cachexies, such as the scorbutic, cancerous, venereal, paludal, saturnine, mercurial, &c., of a cachexy specially belonging to the insane, which may be properly considered as the result of the latent progress of mental alienation and of its continuous action on the animal economy, aided or not by the concurrence of certain particular hygienic conditions.

*Symptoms.*—It does not require a long observation of the insane, in order to recognise that some in whom the disorder has passed to the chronic state, end by presenting an alteration in nutrition, the progress of which, though very variable, is ordinarily slow. This alteration betrays itself in the first instance by a certain emaciation, by the tendency to diarrhœa, and by a successive diminution of strength; but that which appears to me to constitute one of its essential characteristics is the tendency presented by the skin of those individuals who are, or are about to be, affected by it, to undergo various changes; in fact, it is the pathological result depending upon the relation which exists between the dermis and the nervous system. In many instances alterations of the skin which appear to us very variable in their nature coincide or alternate with disorders of the digestive apparatus. At times, and most frequently under the influence of insolation, there may be observed on the dorsal surface of the hand, and extending more or less up the fore-arm, erythematous symptoms, uniting all the characters assigned by writers on the subject to pellagrous erythema, from scarlet erythema to blackish erythema, accompanied with exfoliation; the back of the feet, the neck and the breast, when they remain uncovered, sometimes participate in this state. For an *exposé* of the symptoms of the progress and other points in the history of this form of the cachexy of insanity, answering to that which we have hitherto described by the name of *a variety of pellagra proper to the insane*, we must refer the reader to our preceding memoirs. That which completes the analogy between this form of the cachexy of insanity and pellagra is the period of evolution and habitual exacerbation of its symptoms, and their amendment or almost complete remission after this period. At times, there is observed, as in the last stage of pellagra, scorbutic spots, œdema of the extremities, a general earthy appearance of the skin; at other times, as we have observed in two cases, and as M. Bonacossa, the eminent chief physician of the asylum at Turin tells me he has also observed in his practice, this membrane assumes a bronze tint which recalls the malady known as Addison's *bronzed skin*. In some cases we may observe eruptions successively vesicular, papulous, squamous and furuncular, constituting a sort of lichen (poussée), as noticed by M. Girard of Auxerre. Neither is it very uncommon to see *dartres farineuses*, most frequently on the face, and sometimes affecting circular forms with almost geometrical regularity. We have twice observed purpuric eruptions over the whole body. In two cases we have seen the skin assume the characteristics of *psoriasis diffusa*, and M. Baume of Quimper has told me that he once made a similar observation. Several bullæ of pemphigus have also been observed by us in the course of the cachexy which we are now considering. We believe we may refer to alterations of the cutaneous organs, sometimes observed in the case of lunatics threatened with, or actually in the state of cachexy, a certain hypertrophy with deformity and blackish discoloration of the nails, to which our esteemed friend, M. le Dr. Payen of Orleans, has particularly called our attention. Perhaps, also, the bloody tumours of the pavilion of the ear, to which our dear and venerated master, M. Ferrus, was the first to call attention in his lessons at Bicêtre, and which have recently been the object of an interesting monograph by M. Foville, jun., are not unconnected with the general disposition which we are now describing.

Most frequently the skin of cachectic lunatics, or those who are about to become so, is dry, rough, and does not perspire. Finally, in some cases no appreciable alteration in the skin is to be noticed.

With reference to these alterations of the skin, which so evidently testify to a pathological relation between the dermis and the nervous system, we ought to allude incidentally to a fact observed by our colleagues as well as by ourselves, that is, the relative frequency of erysipelas and ichthyosis amongst the insane. We do so, however, without referring it to the cachexy now under consideration.

When the cachexy of the insane threatens to assume the character of pellagra it is not uncommon, as in the latter disorder, to see the cutaneous symptoms long precede the development of the cachexy: it is often, in fact, only after a long series of vernal exacerbations, and at the end of several years, that the cachectic state really commences. One of the three cases which M. Baillarger presented in our name to the Academy of Medicine, that of the patient named Bureau, has only been cachectic for one year, or speaking more correctly, it is only one year since the alteration of nutrition began to operate in him, and it has progressed but very slowly.

The cutaneous symptoms which we have enumerated almost always accompany the cachexy of insanity, but their intensity does not always measure its degree; thus, for example, they are sometimes wanting in the last stage of the affection, although they may be very decided in its commencement, and before any appearance of emaciation; they may also be wanting, as we have already said, during the whole course of the cachexy. It is not so with its other characteristics, marked by disorders of the digestive apparatus and nervous system, which are common to all forms, and rarely vary except in degree.

Among the digestive symptoms which characterize this special cachexy the most prominent is without doubt diarrhœa. This diarrhœa is often colliquative and incoercible; the stools are generally serous, sometimes bilious, very rarely bloody, and are scarcely ever accompanied with colic. The belly is supple, painless, and ordinarily depressed. What distinguishes this diarrhœa is that throughout its continuance the tongue is ordinarily clean and humid, the appetite is maintained, and the apyrexia is perfect. In some cases, however, and especially in the last stage, the tongue is dry and horny, the thirst raging, and the appetite gone; but these cases are, we repeat, exceptional.

Being rather an asthmic flux than a symptom properly so called of enteritis, the diarrhœa of our cachectics sometimes ceases after a duration varying from a few days to as many months, and it always exhibits a strong tendency to reproduce itself, without any other assignable cause, in most instances, than a slight increase in the general asthenia. This tendency to relapse and the incoercibility augment as the patient approaches the last stage of the cachexy of insanity, and there are few cases in which they do not mark the ultimate step.

In certain cases cachectic lunatics are the prey of burning thirst; some, especially those whose condition partakes of all the characters of pellagra, complain of a burning sensation in the epigastrium of the nature of pyrosis. Ptyalism often exhibits itself; in which case the buccal mucous membrane is red, tumefied, sometimes aphthous, and the papillæ of the tongue are generally depressed.

The urinary secretion presents no special modification; we have never discovered in it any trace of albumen or saccharine element, its reaction has always appeared more or less acid. In some cases, however, the acidity of the urine has seemed to us very notably diminished; the secretion is most commonly limpid pale and free from sediment.

We here offer these remarks only upon the urinary secretion in the cachexy of insanity, reserving to ourselves their completion by ulterior researches, and if need be their rectification.

Nothing particular has been observed with reference to the heart and lungs; the latter less frequently become tuberculous than might have been expected; but we believe we can trace to the cachexy of insanity certain cases of venous obstructions and arterial obliteration followed by sphacelus of entire members, which we have sometimes had occasion to observe. In general, whatever may be the stage of this cachexy, no *bruit de souffle* is perceived in the carotids; a diminution of caloricity may, however, generally be observed, as also a diminutiveness and a characteristic depression of the pulse.

At the same time that the symptoms we have enumerated develope themselves, general enfeeblement progresses until death, which is ever the fatal result, ensues.

In some of our cachectics we observe a certain curvature of the vertebral column, although the patients do not ordinarily complain of any pain in that region; this symptom is particularly marked in one of our pellagrous patients at the asylum of Sainte-Gemmes, who for some years has also exhibited some choreic symptoms.

We have never observed at any period in the cachexy of insanity any symptoms of general or special paralysis which we could refer to a lesion of the nervous centres, and in particular to the spinal chord; nevertheless as we have remarked in a previous paper, the general muscular debility sometimes calls to mind that which characterizes the general paralysis of the insane.

In proportion as the cachexy displays itself and becomes more pronounced the dementia becomes confirmed.

*Progress.*—The progress of the cachexy of insanity is properly speaking continuous: intermission and abatement are at times observed, but they only relate to certain of the symptoms, for example, the cutaneous and digestive symptoms, which when they exist generally pursue the same evolution as the symptoms of pellagra.

Although latent, then, the progress of the cachexy is no less real; at most it can only be said that the malady is sometimes stationary.

The mode of inversion is variable; thus in those cases in which the affection assumes the characters of pellagrous cachexy, the cutaneous symptoms may precede for a long time, many years, for example, the development of the cachectic state, and there is then recognised in the progress of the malady the two periods that we have admitted; the first extending from the apparition of the earliest cutaneous or other symptoms to the moment when the constitution receives a cachectic impress, which we have termed *pellagrous properly so called:* the second extending from the commencement of the cachexy to the termination of the affection. In those cases, on the contrary, in which the cachexy manifests itself without having been preceded by any cutaneous or other symptom, it is evident that there is but one period.

It follows from the exposition we have given of the symptoms of the cachexy of the insane that, as in pellagra, the modification in nutrition which characterizes the affection is most ordinarily preceded by a group or a succession of disorders in the cutaneous system, the digestive apparatus, and the nervous system. The cutaneous symptoms, it is true, sometimes fail; but as (still as in pellagra) they constitute but a symptomatic expression of a general interior disposition, and this symptomatic expression can fail, and indeed very often fails, without the aforesaid disposition the less existing, a conclusion cannot be drawn from their absence contrary to the opinion which we have expressed.

This is the place, moreover, to consider that it is much less in real and effective changes that the symptoms derived from the skin of our cachectics consist, than in a disposition of this membrane, in a sort of aptitude, so to speak, to become morbidly changed in divers fashions under certain conditions, just as is observed in pellagrous individuals. We know indeed that insolation ordinarily constitutes the essential condition of the development of alterations of the skin among the pellagrous as among our cachectics, and we are able, in removing patients from this influence, to prevent effectively the alterations; but it is evident that in this case, if the skin is not altered, it does not lack the morbid aptitude to become changed, and that just as, according to the first Sydenham, there is *variola without eruption*, so there may be pellagra and cachexy of the insane without cutaneous symptoms.

The inquiry which we have instituted in the majority of our principal asylums, with the kind aid of our honourable fellow-labourers, has fully confirmed these last-named data. It results, in effect, that notwithstanding cachexies inherent to the progress of mental alienation exist in every establishment, in some, nevertheless, these cachexies are not accompanied with any special alteration of the skin; it results also that in those cases where the alterations of the skin are manifestly linked to the progress of the cachexy peculiar to the insane, these alterations have not presented everywhere the same characters, witness, for example, the asylums of Auxerre and of Toulouse. The conclusion to be drawn from this difference is that, if the state of mental alienation is the primary condition of the development of the cachexy of the insane, this cachexy, nevertheless, is dependent for its manifestations upon certain hygienic conditions peculiar to each medium.

*Differential Diagnosis.*—The special conditions under which the cachexy we have under consideration is developed, prevent us from confounding it with other cachexies, hence it is not necessary to detail the differential diagnosis. We think, however, that it is of importance to note that this cachexy ought to be distinguished from the marasmus which terminates the course of general paralysis, and with which it has nothing in common. It is well also not to confound the cachexy with the modifications of nutrition which accompany divers maladies that may supervene incidentally during insanity. It is manifest, for example, that it is necessary to distinguish the cachexy of the insane from the modification of nutrition in a lunatic, which depends upon the development of incidental tubercular phthisis.

*Pathogeny and Etiology.*—The primary condition of the development of the cachexy of the insane being the state of mental alienation, it follows from this that its etiology will not be doubtful; as to its pathogeny it will be allied to the mechanism of this cause, and we cannot but reproduce here what we have previously said apropos of our variety of pellagra, and of which the application is equally fitted to the cachexy which we now study:—

"If it be true, and no one doubts it, that an emotion, a chagrin, a pleasure, any moral cause, in short, can exercise an influence upon the play of the organic functions, and produce a modification, of whatever kind, of the general health, it ought also to be true, *à fortiori*, of mental alienation, that exaggeration, that *ne plus ultra* of all moral causes. This follows, moreover, from a physiological principle which appears to me to have the force of a law, to wit, that the general health may be regarded as the result of a pre-established repartition of the innervation between all the organs. Every circumstance which disturbs the exercise of a function, in a manner to augment or diminish the quantity of innervation which physiologically devolves to it, ought to induce a change in the general repartition, a rupture in the equilibrium, and consequently a disordered state of the health.

"It follows from this that mental alienation, by the disturbance of the cerebral functions which characterize it, ought, in modifying the innervation almost at its source, to lead much more certainly than any other disturbing cause to the rupture of equilibrium just referred to, and, in consequence, to a change in the general health. Now, we believe that pellagra is in the number of these possible effects."

This being presumed, it is easy to admit that the chance of the cachexy under consideration being produced, will be greater in proportion to the older date of the mental alienation. All things being equal, observation demonstrates that, of all the forms of mental alienation, that which most disposes to the cachexy is the melancholic or depressive.

Of 65 lunatics suffering from or threatened with the peculiar cachexy, we observed in 18 lypemania with stupor or depression; in 32 lypemaniacal dementia, also with stupor or depression; in 5 chronic mania; in 2 dementia, consecutive to epilepsy; and in 7 idiocy.

It results from these data that it is depression which plays the principal part in the production of the cachexy of the insane. It is always important, in order to appreciate the comparative influence of depression and excitation, to hold count of the very marked predominance which exists in the asylums in which we collected our observations of the melancholic form of mental alienation, but as the cachexy of the insane, when it assumes a pellagrous character, induces depression, that is to say converts mania into lypemania among those individuals who are attacked with the malady, it results that the portion of maniacs who suffer from the said cachexy is greater than at first appears. *A priori*, it does not appear that it could be otherwise, because prolonged mania brings about an expenditure of innervation, which necessarily, sooner or later, leads to exhaustion, the effects of which, although not invariably manifested during the progress of the expenditure, are not the less real, and in the end appreciable. Also, when the source of innervation is exhausted among maniacs, in consequence of the excessive expenditure it has been subjected to, it is common to see depression succeed hastily to excitation, and the marasmus progresses much more rapidly than in other cases. We have seen recently a striking example of the rapidity with which patients sink in these circumstances. It happened in the case of one of our boarders, the sister of a physician, attacked with mental alienation characterized by a delirium of persecutions and habitual exaltation. Her physical health had always been sustained without the appearance of exhaustion, when, fifteen days before death, she began to exhibit the first symptoms of an enfeeblement, the progress of which was most rapid. During these fifteen days, in proportion as the marasmus became more conspicuous, the excitation gave place to depression, but not without reappearing at certain intervals, as if to complete the exhaustion of its source even to death, which seemed to be the result of a last glimmer of exaltation.

It will be understood that the cachexy of the insane, when it pursues a progress so rapid, is not marked by the same characters, and is much more rarely accompanied by symptoms recalling more or less pellagra, than when it pursues, as among melancholics, a slower progress.

This difference in the characters of the cachexy of the insane, according as the progress is more or less rapid, or according as it affects patients in a state of depression or exaltation, explains, perhaps, the differences observed, in this respect, in the asylums of the department of the Seine, where the maniacal form of alienation predominates over the melancholic, and in the majority of the provincial asylums where the contrary is ordinarily observed. It is possible also, that under the same relation there are notable differences between the asylums of the North, South, East and West.

The influence of mental alienation upon the development of a special cachexy can be heightened or diminished, and sometimes altogether neutralized, by certain hygienic conditions. It is a fact, recognised at first sight, that in the *maisons de santé*, and in the boarders' quarters of public asylums, where the regimen is more comfortable than in the pauper sections, the development of the special cachexy is less manifest, and when it supervenes, it never assumes a pellagrous character.

We have, in certain cases, retarded and even stopped the progress of the special cachexy by a simple modification in the regimen; for example, in rendering it more substantial and more nutritious. The use of wine has seemed to us to exercise a prophylactic influence. This is to be gathered plainly from the following observation:—Until the 1st of January, 1859, our lunatics received wine but once or twice weekly at most, and from the 4th of June, 1854, to that period—that is to say, during a period of four years—we had recorded 66 cases of the affection that we have described under the name of a *variety of pellagra peculiar to the insane*, in a total of 1287 individuals, who made part of the population in the interval. Now, since the 1st of January, 1859, the distribution of wine has been daily, and during the course of the year we have not observed any new case of this variety of pellagra.

We cannot terminate our remarks on the etiology of the cachexy of the insane, and in particular of its pellagrous form, without saying a few words on the argument which some physicians have deduced from this affection against the opinion which is maintained by several that the use of maize, deteriorated or not by *verdet*, is the essential cause of pellagra. Now, although we rank ourselves among the adversaries of this opinion, we do not think that the existence of a variety of pellagra peculiar to the insane and consecutive to mental alienation proves anything against the etiological hypothesis referred to, from the moment when this variety of pellagra is regarded as of a distinct and every way special species. It might be, after all, although this is but another hypothesis, that, if the influence of deteriorated maize was as real as some physicians believe, it would act by producing in the nervous system previous perturbations, of which the effects upon the nutrition are analogous to those which result from the continued action of mental alienation. This opinion would be all the more admissible since, among the nervous perturbations which characterize pellagra properly so called, mental alienation is observed in the end, and in its melancholic form.

I may be permitted here, to express the opinion that the practice indicated by one of the most fervent adepts of Balardini, of submitting the maize, before delivering it for consumption, to torrefaction by the process of Bourguignon, should be undertaken sooner or later. I am an adversary, as I have already said, to the theory that the use of maize is the sole cause of pellagra, but not a bigoted one. Were it but for the sake of dissipating the obscurity which still invests the etiology of pellagra, I think that the foregoing experiment is desirable.

[Dr. Billod in terminating his paper dwells briefly on the prognosis, pathological anatomy, and treatment of the cachexy he describes. The *prognosis*, as would be surmised, is very grave; *pathological anatomy* does not throw any definite light on the nature of the cachexy, but Dr. Billod is inclined to think general or partial softening of the spinal cord has some connexion with the cachexy, particularly its pellagrous form; finally, the *treatment*, apart from that which is proper to the form of mental alienation which precedes the cachexy, is simply hygienic.]

